# Lensless Quantitative Phase Imaging with Bayer-Filtered Color Sensors Under Sequential RGB-LED Illumination

**DOI:** 10.3390/jimaging12030101

**Published:** 2026-02-26

**Authors:** Jiajia Wu, Yining Li, Yuheng Luo, Leiting Pan, Pengming Song, Qiang Xu

**Affiliations:** 1 School of Physics, TEDA Institute of Applied Physics, Nankai University, Tianjin 300071, China; 2 Guangzhou National Laboratory, Guangzhou International BioIsland, Guangzhou 510005, China; 3 Collaborative Innovation Center of Extreme Optics, Shanxi University, Taiyuan 030006, China

**Keywords:** lensless on-chip microscopy, Bayer-filtered color sensor, quantitative phase imaging, polychromatic phase retrieval

## Abstract

Lensless on-chip microscopy enables high-throughput, wide-FOV imaging; however, the Bayer color filter array (CFA) in standard color sensors spatially multiplexes spectral channels, introducing sub-sampling and spectral crosstalk that degrade phase retrieval. We propose a Wirtinger Poly-Gradient Solver (WPGS) for quantitative phase reconstruction with Bayer-filtered color sensors under sequential Red–Green–Blue Light-Emitting Diode (RGB-LED) illumination. The method combines Transport of Intensity Equation (TIE)-based initialization with polychromatic Wirtinger optimization to suppress CFA-induced artifacts and enable pixel super-resolution (PSR). Experiments resolve a 2.76 μm linewidth using a 1.85 μm pixel-pitch sensor, exceeding the nominal Nyquist limit imposed by pixel sampling. We further demonstrate label-free imaging of HeLa cells and unstained tissue sections, supporting high-throughput digital pathology and offering potential for longitudinal biological observation.

## 1. Introduction

While indispensable for life sciences [1,2], conventional microscopy is fundamentally constrained by the field of view (FOV) versus resolution trade-off, dictated by physical invariants such as optical étendue and the space-bandwidth product (SBP) [3,4,5]. In practice, achieving high resolution typically requires high-numerical-aperture objectives, which inevitably restrict the FOV and limit throughput in applications such as large-scale cell monitoring and comprehensive histological analysis [6,7,8].

Lensless on-chip microscopy addresses this limitation by placing the specimen in close proximity to the image sensor and reconstructing the complex field computationally from recorded diffraction patterns [9,10]. Despite its compact form factor and large FOV, many high-performance lensless systems still rely on monochromatic detection [11,12]. Standard color CMOS sensors are attractive due to their accessibility and low cost, yet the Bayer color filter array (CFA) spatially multiplexes spectral channels, introducing sub-sampling and spectral crosstalk [13,14]. These effects reduce the effective spatial-frequency support and induce model-mismatch errors in phase retrieval [15,16], resulting in chromatic artifacts and degraded reconstruction fidelity [17,18].

Existing mitigation strategies typically trade hardware simplicity for spectral efficiency, for example, by physically removing the CFA or operating on a single color channel [19,20]. Moreover, conventional demosaicing methods—largely optimized for human visual perception—tend to attenuate the high-frequency oscillatory fringes that are essential for coherent diffraction imaging. To overcome these limitations without modifying standard sensors [15,16,17], we introduce the Wirtinger Poly-Gradient Solver (WPGS), a multi-spectral computational framework for quantitative phase recovery. The key contributions of this work are summarized as follows: (i) We propose a physics-based polychromatic model to numerically decouple the periodic CFA modulation; (ii) We develop a three-stage iterative engine that progressively unlocks sub-pixel structural details; (iii) We demonstrate that by leveraging spectral diversity as a strong physical constraint, the space-bandwidth product (SBP) can be effectively extended on commercial Bayer-filtered sensors.

By combining sequential Red–Green–Blue Light-Emitting Diode (RGB-LED) illumination with this iterative framework, WPGS suppresses crosstalk [21] and enables pixel super-resolution (PSR). Experiments successfully resolve a 2.76 μm linewidth using a sensor with a 1.85 μm pixel pitch, exceeding the nominal Nyquist limit. We further demonstrate label-free imaging of HeLa cell populations and unstained tissue sections, offering significant potential for longitudinal live-cell monitoring and digital pathology.

## 2. Materials and Methods

In this section, we present the hardware and algorithmic components of our lensless imaging system. The process involves sequential RGB illumination, adaptive demosaicing, and a multi-spectral iterative reconstruction to achieve quantitative phase recovery from Bayer-filtered holograms.

### 2.1. Experimental Configuration and Sequential Illumination

The imaging system is a compact lensless on-chip microscope designed for scan-free quantitative phase acquisition. Diffraction patterns are recorded using a Sony IMX226 CMOS sensor (Sony Corporation, Tokyo, Japan) (12.4 MP, 1.85 μm pixel pitch). The sample is placed at a sensor distance of approximately 1 mm to balance space-bandwidth product and fringe contrast. Illumination is provided by an integrated RGB-LED module with center wavelengths of $\lambda_{B}=460\;\mathrm{nm}$, $\lambda_{G}=520\;\mathrm{nm}$, and $\lambda_{R}=620\;\mathrm{nm}$ (Full Width at Half Maximum, FWHM ≈30 nm). The three LEDs are triggered sequentially to acquire a multi-wavelength hologram stack within seconds, providing spectral diversity for the subsequent reconstruction.

### 2.2. Biological Specimen Preparation and Comparative Imaging

To assess performance on biologically relevant specimens, we imaged fixed HeLa cells and unstained mouse kidney tissue sections. HeLa cells were cultured in Dulbecco’s Modified Eagle Medium (DMEM) supplemented with 10% fetal bovine serum and 1% penicillin–streptomycin (Gibco, Carlsbad, CA, USA) at 37 °C in 5% CO_2_. Cells were seeded on glass-bottom dishes (NEST, Wuxi, China) and fixed at 70–80% confluency using 4% paraformaldehyde (Biosharp, Hefei, China), followed by rinsing with phosphate-buffered saline. Unstained mouse kidney tissue slices (Tianjian, Xinxiang, China) were deparaffinized and rehydrated prior to imaging. For lensless acquisition and quantitative analysis, the sample was maintained in phosphate-buffered saline (PBS), which was treated as the surrounding medium in subsequent dry-mass estimation. For reference, corresponding fields of view were acquired using a commercial inverted microscope (Axio Observer A1, Carl Zeiss AG, Oberkochen, Germany) with phase-contrast objectives (5× and 10×, 0.4 NA). These images were used as a reference to evaluate the reconstruction fidelity of WPGS.

### 2.3. Forward Modeling of Polychromatic Bayer-Filtered Holography

In practical on-chip microscopy, the monochromatic assumption is violated by the finite spectral bandwidth of LED sources, leading to polychromatic averaging and wavelength-dependent propagation. For the Sony IMX226 sensor, the recorded intensity $I_{c}$ for channel c∈{R,G,B} is modeled as an integral over wavelength, weighted by the illumination spectral power distribution (SPD) and the combined CFA–sensor spectral response:

(1) $$I_{c}\left(x,y\right)=\int_{\lambda }S_{c}\left(\lambda \right)\;\cdot \;R_{c}\left(\lambda \right)\;\cdot \;{\left|P_{z}\left\{U_{obj}\left(x,y\right);\lambda \right\}\right|}^{2}d\lambda .$$

Here, $S_{c}\left(\lambda \right)$ represents the approximately Gaussian SPD of the *c*-th LED, and $R_{c}\left(\lambda \right)$ denotes the combined spectral response of the CFA–sensor system (incorporating both CFA transmittance and sensor quantum efficiency) for channel *c*. The operator $P_{z}$ defines free-space propagation based on the angular spectrum method.

### 2.4. Adaptive Demosaicing and Multi-Spectral Registration

Because the Bayer CFA spatially sub-samples each color channel, conventional demosaicing can introduce interpolation artifacts. We adopt an edge-aware strategy where horizontal and vertical gradients ($G_{h},G_{v}$) determine the interpolation direction:

(2) $${\hat{I}}_{\mathrm{pixel}}=\left\{\begin{array}{cc} \frac{1}{2}\left(I_{left}+I_{right}\right), & |G_{h}\left|<\right|G_{v}|\\ \frac{1}{2}\left(I_{up}+I_{down}\right), & |G_{h}\left|>\right|G_{v}| \end{array}\right.$$

Ties are resolved by averaging both directions. Following demosaicing, wavelength-dependent lateral shifts are compensated using Discrete Fourier Transform (DFT)-based registration to enforce spatial correspondence across multi-wavelength channels.

### 2.5. The WPGS Reconstruction Pipeline

**Figure 1:**
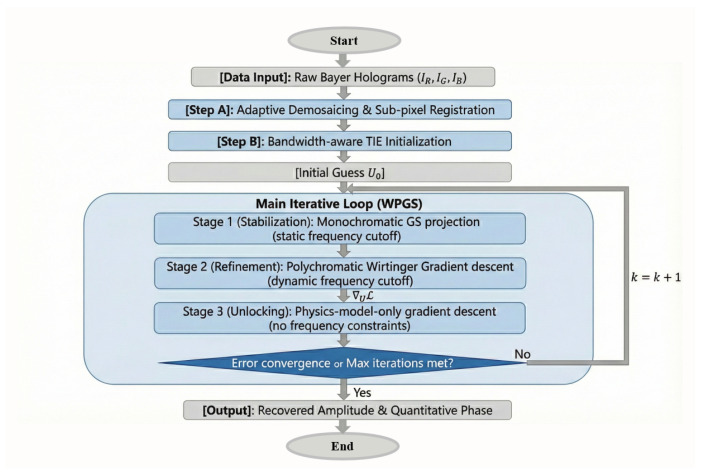
Schematic workflow of the WPGS algorithm. The framework integrates sequential RGB acquisition, deterministic TIE initialization, and a three-stage iterative engine—stabilization, polychromatic refinement, and resolution unlocking—to enable PSR phase reconstruction.

The WPGS framework addresses the inherent low-frequency sensitivity of Transport of Intensity Equation (TIE) and the high-frequency convergence challenges of iterative phase retrieval. The logical flow of the algorithm is detailed in Figure 1.

#### 2.5.1. Phase Initialization via Bandwidth-Aware TIE

The reconstruction pipeline is initiated with a deterministic phase estimate derived from a Fast Fourier transform (FFT)-based TIE solver [22]. To circumvent the requirement for physical axial scanning, we adopt a heuristic spectral-to-axial mapping that approximates the axial intensity derivative by leveraging the intensity contrast between disparate spectral channels. To account for polychromatic decoherence and compensate for the inherent spectral responsivity mismatch between color channels, we introduce a bandwidth-aware correction factor γ:

(3) $$\frac{\partial I}{\partial z}\approx \gamma \;\cdot \;\frac{I_{\lambda_{1}}-I_{\lambda_{2}}}{\Delta z_{\mathrm{eff}}},$$

where $I_{\lambda_{1}}$ and $I_{\lambda_{2}}$ denote the holograms captured under two distinct center wavelengths. The parameter γ is a bandwidth-aware correction factor that also serves as a dynamic step-size during the optimization (decaying linearly from 0.5 to 0.1), and $\Delta z_{\mathrm{eff}}=z_{0}\;\cdot \;\left(\Delta \lambda /\lambda \right)$ represents the equivalent axial defocus separation facilitated by wavelength diversity via Fresnel scaling. Here, $z_{0}$ is the sample-to-sensor distance, and λ is the center wavelength of the primary imaging channel. To compensate for the global piston ambiguity and systematic gain attenuation inherent in TIE-based solvers, we apply a linear calibration $\Phi_{\mathrm{cal}}=a\Phi_{\mathrm{rec}}+b$ only in numerical simulations where a ground-truth phase is available, prior to quantitative evaluation and visualization. For experimental results, we do not rescale the phase values; instead, we only perform median-background piston removal and sign normalization as described in Section 2.6.

#### 2.5.2. Non-Linear Refinement via Wirtinger Poly-Gradient Optimization

The initial estimate is refined through Wirtinger optimization [23]. The objective function compares measured amplitude with band-integrated predictions:

(4) $$L\left(U\right)=\sum_{c\in \{R,G,B\}}{∥\sqrt{I_{c}^{\mathrm{meas}}}-{\left(\sum_{s=1}^{S}w_{c,s}{\left|P_{z}\left\{U;\lambda_{c,s}\right\}\right|}^{2}\right)}^{\frac{1}{2}}∥}_{2}^{2}$$

In Equation (4), *U* represents the complex amplitude of the specimen to be recovered, and $P_{z}\left\{\;\cdot \;;\lambda \right\}$ is the free-space propagation operator at wavelength λ. The nonnegative weights $w_{c,s}$ satisfy $\sum_{s}w_{c,s}=1$ and are computed as $w_{c,s}\propto S_{c}\left(\lambda_{c,s}\right)R_{c}\left(\lambda_{c,s}\right)\Delta \lambda$, where $S_{c}$ and $R_{c}$ denote the source emission spectrum and the Bayer filter’s spectral response, respectively. Additionally, *S* denotes the number of discrete spectral sampling points (set to S=3 to balance reconstruction fidelity and efficiency).

To ensure convergence and sub-pixel accuracy, the optimization is executed for 100 total iterations, divided into three structured stages corresponding to the flowchart in Figure 1: Stage 1 (Iterations 1–20): Focuses on monochromatic stabilization. By using monochromatic projections with a static frequency cutoff, we stabilize the initial phase estimate and suppress coarse noise. Stage 2 (Iterations 21–70): Introduces the polychromatic Wirtinger gradient with a dynamic frequency cutoff. To ensure robust convergence, the step size is linearly decayed from 0.5 to 0.1, effectively decoupling the CFA-induced crosstalk. Stage 3 (Iterations 71–100): Performs final resolution unlocking. By removing all frequency constraints, the physics-model-only descent recovers high-frequency diffraction details and achieves final pixel super-resolution. The computational efficiency of the WPGS framework is optimized for high-throughput applications. In our implementation using graphics processing unit (GPU) acceleration (NVIDIA RTX 3090), a typical 512×512 pixel reconstruction with 100 iterations requires approximately 3.4 s, making it suitable for rapid biological screening and live-cell monitoring.

### 2.6. Quantitative Dry-Mass Estimation

For lensless phase retrieval, the recovered phase contains a global piston ambiguity. Therefore, a baseline correction was applied by subtracting the median phase measured in a non-cell background region. A sign convention was subsequently enforced by flipping the phase map when the mean phase within the segmented cell region was negative, ensuring that cellular regions exhibit positive optical path delay. For dry-mass quantification, a whole-mask integration strategy was adopted. To suppress residual negative contributions caused by noise or slight model mismatch, we set negative phase values inside the cellular mask to zero before integration, i.e., $\phi_{+}\left(x,y\right)=\max\left(\phi \left(x,y\right),0\right)$. Note that this non-negative truncation is applied only for dry-mass integration; phase maps are visualized using the piston-corrected and sign-normalized phase ϕ(x,y) without truncation. The total dry mass within the segmented cellular region Ω is estimated as [24,25]:

(5) $$M=\frac{\lambda }{2\pi \alpha }\int_{\Omega }\phi_{+}\left(x,y\right)\;dA$$

where $\phi_{+}\left(x,y\right)$ denotes the piston-corrected and sign-normalized phase (in radians) after non-negative truncation, λ is the effective wavelength (520 nm, Plan A), α is the refractive index increment (0.18–0.20 mL/g), and dA is the pixel area. The estimated mean dry mass per cell is reported as M/N, where *N* denotes the number of segmented cells within the ROI. For reproducible visualization of the experimental phase maps, all phase maps are displayed in radians using a symmetric dynamic range [−m,m], where *m* is determined by the 99th percentile of |ϕ(x,y)| within the displayed ROI.

## 3. Results

In this section, we evaluate the performance of WPGS by first validating the forward model and noise robustness in numerical simulations, and then assessing practical imaging performance in experiments.

### 3.1. Numerical Simulation and Quantitative Evaluation

**Figure 2:**
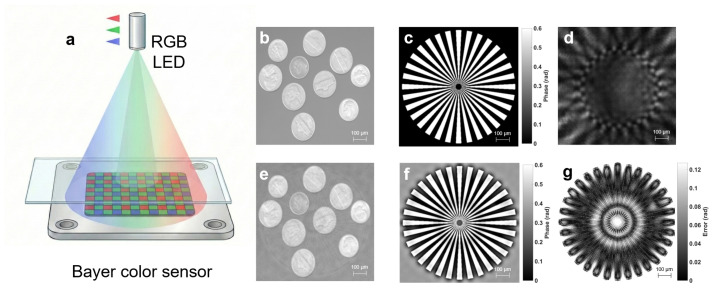
Numerical validation of the WPGS framework. (**a**) Lensless on-chip imaging geometry used in simulation. (**b**,**c**) Synthetic ground-truth amplitude and phase. (**d**) Simulated raw hologram under green-channel illumination. (**e**,**f**) Reconstructed amplitude and calibrated phase using WPGS. (**g**) Absolute phase error map $|\Phi_{\mathrm{cal}}-\Phi_{\mathrm{gt}}|$ (in rad) with an independent color scale.

Numerical simulations using a complex phantom validated the reconstruction fidelity under configurations matched to Section 2.1, with a noise surrogate introduced for experimental perturbations. Specifically, the simulation employed a 512×512 complex phantom with a pixel size of 1.85 μm. We modeled the polychromatic illumination using the measured spectral power distributions (SPD) of RGB LEDs and introduced a Gaussian noise surrogate (std = 0.01) to simulate experimental perturbations. Compared to the blurred raw hologram (Figure 2d), the WPGS framework significantly improved structural preservation, achieving an amplitude Root Mean Square Error (RMSE) of 0.0229 and a phase RMSE of 0.0531 rad within the strict object ROI after linear calibration (corresponding to a relative error of approximately 8.9% with respect to the 0.6 rad phase peak). The phase Structural Similarity Index (SSIM) increased from 0.4751 (the TIE-only baseline) to 0.5835 post-optimization. To compensate for visualization consistency and systematic gain attenuation inherent in TIE-based solvers, a linear calibration $\Phi_{\mathrm{cal}}=a\;\Phi_{\mathrm{rec}}+b$ was applied within the effective object ROI prior to visualization and quantitative evaluation. Specifically, the phase residual map (Figure 2g) confirms that absolute errors remain below 0.1 rad for 93.86% of pixels within the strict object ROI (approximately 40.8% of the full field, calculated by counting pixels with an absolute error less than 0.1 rad relative to the total pixel count), with minor deviations localized at high-frequency boundaries where recovery is ill-conditioned due to Bayer sub-sampling limits. The residual in Figure 2g is visualized as $|\Phi_{\mathrm{cal}}-\Phi_{\mathrm{gt}}|$ in radians (no normalization); therefore, its dynamic range is intentionally much smaller than that of the phase maps in Figure 2c,f. The absence of periodic checkerboard artifacts validates effective CFA decoupling and robust fine-scale phase recovery. Although some minor grid-like artifacts are visible in the reconstructed amplitude (Figure 2e), they are primarily attributed to the inherent spatial sub-sampling of the Bayer CFA. Crucially, these artifacts have a negligible impact on the quantitative phase accuracy, as the phase residual remains extremely low (Figure 2g). This spatial error distribution confirms the physical consistency of our model: the framework excels in stabilizing bulk phase retrieval, while the localized boundary deviations highlight the inherent trade-off between sub-pixel resolution and the discrete sampling limits of the Bayer CFA. It is important to acknowledge the inherent limitations of the proposed framework at this stage. While WPGS significantly enhances phase fidelity, its performance is subject to a dual constraint: first, the physical information loss caused by the Bayer CFA occlusion limits the recovery of extreme high-frequency components; and second, the iterative solver faces inherent challenges in maintaining convergence stability when reconstructing sharp, non-continuous phase transitions from sub-sampled data. Recognizing these boundaries is crucial for further optimization of the sampling and reconstruction engine.

### 3.2. Experimental Performance and Resolution Analysis

**Figure 3:**
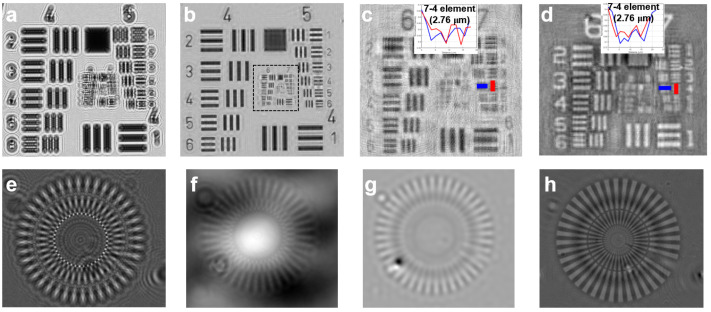
Experimental characterization and resolution analysis. (**a**) Raw diffraction pattern (green channel, $\lambda_{G}=520$ nm) of the USAF 1951 target. (**b**) Full FOV amplitude reconstruction. (**c**) Magnified amplitude region and (**d**) phase reconstruction resolving Group 7 Element 4 (2.76 μm linewidth); the colored lines indicate the line-scan locations used to extract the corresponding intensity profiles (shown as insets) for resolution assessment. (**e**) Raw diffraction pattern of the Siemens star target. (**f**–**h**) Comparison of phase recovery performance: (**f**) TIE-only, (**g**) monochromatic iterative, and (**h**) the polychromatic WPGS method.

We evaluated the practical imaging performance of WPGS using a USAF 1951 target and a Siemens star target under the configuration described in Section 2.1. Figure 3a shows the raw diffraction pattern of the USAF target, which is affected by Bayer CFA modulation and exhibits mosaic artifacts and diffraction-induced blur.

The full FOV amplitude reconstruction is shown in Figure 3b, and a magnified region for resolution assessment is provided in Figure 3c. WPGS resolves Group 7 Element 4, corresponding to a linewidth of 2.76 μm. To clearly substantiate this claim, we have enlarged the intensity profile insets in Figure 3c,d. The calculated peak-to-valley ratio is approximately 20%, which indicates resolvability of Group 7-4 under a commonly used bar-target modulation criterion. The consistent resolution of these features in both amplitude and phase reconstructions further confirms the reliability of our PSR performance. For a sensor pixel pitch of 1.85 μm, the nominal Nyquist limit imposed by pixel sampling is 2p=3.7 μm; therefore, resolving 2.76 μm features indicates pixel super-resolution (PSR) by surpassing the nominal sampling limit of a single Bayer-subsampled channel. The corresponding phase reconstruction (Figure 3d) preserves contrast with a stable background across Groups 6 and 7.

Compared with numerical simulations, the experimentally achieved resolution is lower than the best-case prediction (e.g., access to Group 8 elements). This gap is mainly attributable to the Bayer CFA, which imposes periodic sub-sampling and wavelength-dependent crosstalk that attenuate high-frequency fringes at the measurement stage. Under experimental noise, the reduced fringe contrast limits the recovery of sub-pixel structures, thereby constraining the attainable resolution.

To further isolate the contribution of polychromatic optimization, we additionally evaluated phase recovery using a Siemens star target (Figure 3e–h). The TIE-only result (Figure 3f) provides a stable low-frequency estimate but remains strongly blurred, preventing reliable resolution of the inner spokes. The monochromatic iterative reconstruction (Figure 3g) improves apparent sharpness but exhibits pronounced ringing and spatially varying phase inconsistencies, consistent with insufficient constraints to decouple CFA-induced modulation. In contrast, WPGS (Figure 3h) reduces ringing artifacts and yields a more spatially consistent phase map by exploiting spectral diversity under sequential RGB illumination, enabling clearer visibility of spokes toward the center region. This performance leap is inherently rooted in the spectral redundancy provided by the multi-spectral model. Unlike monochromatic baselines that suffer from ill-posed sub-sampling, WPGS leverages the overlapping spectral responses as a physical constraint to effectively resolve high-frequency features beyond the nominal Nyquist limit.

### 3.3. Application to Biological Specimen Imaging

**Figure 4:**
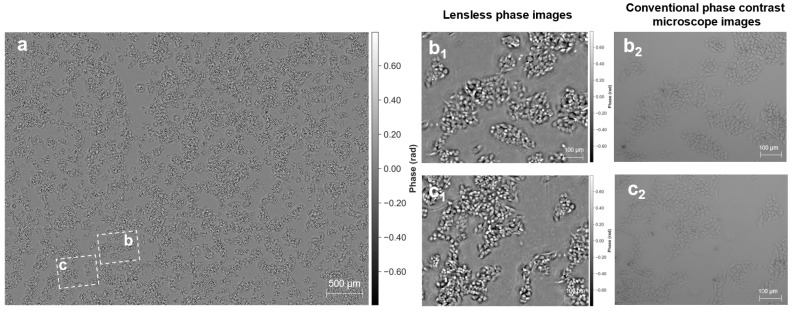
Label-free quantitative phase imaging of HeLa cell populations. (**a**) Wide-field quantitative phase map across the sensor area ($41\;{\mathrm{mm}}^{2}$). (**b1**,**c1**) Magnified lensless phase maps highlighting cellular morphology and intracellular density variations. (**b2**,**c2**) Corresponding reference images were acquired by phase-contrast microscopy (10×, 0.4 NA). All lensless phase maps are shown in radians after median-background piston removal and sign normalization. For reproducible visualization, a symmetric percentile-based dynamic range is used for the displayed phase maps.

We demonstrate label-free quantitative phase imaging on fixed HeLa cells and unstained mouse kidney tissue sections. These specimens exhibit limited contrast in bright-field imaging, whereas the proposed system recovers quantitative phase maps without exogenous staining. Figure 4a shows a full-FOV reconstruction covering approximately $41\;{\mathrm{mm}}^{2}$, enabling visualization of cell distributions across the sensor area. Magnified regions (Figure 4(b1,c1)) reveal cellular morphology and subcellular density variations that are consistent with the corresponding reference phase-contrast images (Figure 4(b2,c2)). To provide a physically interpretable quantitative metric, we report a conservative whole-mask dry-mass estimate for the HeLa-cell ROI acquired in PBS (treated as the surrounding medium). Following median-background piston removal, sign normalization, and non-negative truncation as described in Section 2.6, the total dry mass within the segmented cellular region was estimated as $9.22\times 10^{5}$–$1.02\times 10^{6}$ pg (using α=0.18–0.20 mL/g and λ=520 nm). With N=6462 segmented cells, the corresponding mean dry mass is 142.68–158.53 pg per cell. These values are in line with the reported dry-mass range of mammalian cells measured by quantitative phase microscopy. We emphasize that this whole-mask integration avoids per-cell boundary ambiguity and provides a robust population-level quantitative metric. The phase maps shown in Figure 4 are visualized in radians using a symmetric 99th-percentile-based dynamic range (units: rad), following the same percentile rule described in Section 2.6 for reproducible display.

**Figure 5:**
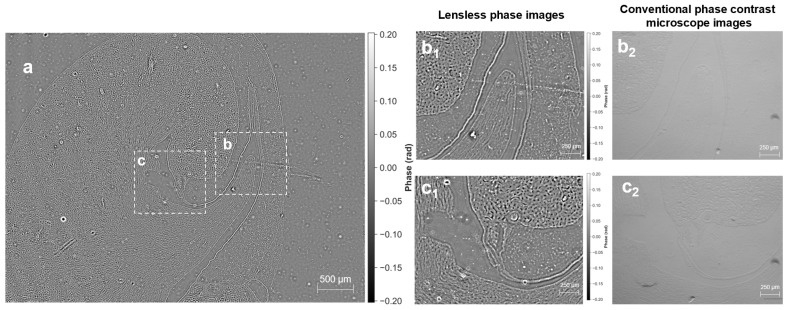
Histological imaging of unstained mouse kidney sections. (**a**) Large-scale quantitative phase reconstruction of the tissue architecture. (**b1**,**c1**) Magnified lensless phase maps resolving glomeruli and convoluted tubules. (**b2**,**c2**) Corresponding reference phase-contrast images for morphological comparison (5×, 0.4 NA). All lensless phase maps are shown in radians after median-background piston removal and sign normalization. For reproducible visualization, a symmetric percentile-based dynamic range is used for the displayed phase maps.

For unstained mouse kidney tissue (Figure 5), histological structures such as glomeruli and convoluted tubules are readily identified. In addition, the reconstructed phase maps show reduced halo artifacts compared with conventional phase-contrast microscopy, leading to a more uniform background across dense tissue regions.

## 4. Discussion

The numerical and experimental results in Section 3 demonstrate that WPGS enables robust lensless quantitative phase imaging with Bayer-filtered color sensors. By combining deterministic TIE-based initialization with polychromatic Wirtinger optimization under sequential RGB illumination, the framework improves reconstruction stability while preserving a scan-free and compact hardware configuration.

### 4.1. Physical Synergy of the Hybrid Framework

The robustness of WPGS stems from integrating complementary mechanisms. TIE provides a deterministic low-frequency phase estimate from the axial intensity derivative, which improves initialization and alleviates low-frequency stagnation and local-minimum trapping in non-linear phase retrieval. Building on this initialization, polychromatic optimization exploits spectral diversity to refine the solution and recover higher spatial-frequency components. The demonstrated sub-pixel enhancement is primarily facilitated by fusing complementary frequency supports across RGB wavelengths. While Section 3.2 presents the quantitative resolution gain, here we emphasize that the framework’s ability to exceed the Nyquist limit is a direct consequence of spectral diversity serving as a strong physical constraint, which numerically mitigates the spatial information loss inherent in Bayer CFA modulation.

### 4.2. Comparison with Alternative Lensless Methodologies

WPGS offers distinct advantages over scanning-based architectures like coded ptychography. While the latter requires extensive frame acquisition and is sensitive to mechanical drift [26,27], our framework achieves multi-wavelength constraints without translational scanning by sequentially switching static RGB LEDs. This configuration significantly enhances temporal efficiency and environmental robustness, rendering the system inherently amenable to prospective longitudinal biological observation without the artifacts associated with mechanical instability. Furthermore, in contrast to monochromatic iterative solvers, the multi-wavelength optimization framework provides intrinsic spectral redundancy across channels. This redundancy facilitates the suppression of background noise, mitigates ringing artifacts, and minimizes cross-channel model inconsistencies, ultimately yielding superior phase reconstruction fidelity.

### 4.3. Error Analysis and Bottlenecks of the Bayer Architecture

Despite the demonstrated resolution improvement, a gap remains between experimental reconstructions and idealized simulations, largely due to the structural constraints of the Bayer CFA. The CFA imposes periodic sub-sampling and wavelength-dependent crosstalk, which attenuate high-frequency diffraction fringes at the measurement stage. While adaptive demosaicing reduces interpolation artifacts, information physically lost due to CFA occlusion cannot be fully recovered computationally. Residual deviations are further exacerbated by partial source coherence and sensor noise, which reduce fringe contrast and increase model mismatch in practical measurements.

### 4.4. Clinical Potential and Future Trajectories

The reconstructions of HeLa cells and unstained tissue sections indicate the potential of WPGS for high-throughput, label-free quantitative pathology. The unit-magnification geometry provides a large SBP, enabling micrometer-scale resolution across the full active sensor area. In addition, the recovered quantitative phase maps reduce halo artifacts commonly observed in phase-contrast microscopy, improving interpretability in dense histological structures. Future work will extend the framework to multi-angle illumination for three-dimensional diffraction tomography and investigate hardware-accelerated implementations for real-time biomedical imaging.

## 5. Conclusions

We presented WPGS for lensless quantitative phase imaging with Bayer-filtered color sensors. By combining bandwidth-aware TIE initialization with three-stage polychromatic Wirtinger optimization, WPGS numerically mitigates CFA-induced crosstalk and enables PSR. Experiments resolved 2.76 μm features over an approximately $41\;{\mathrm{mm}}^{2}$ field of view and demonstrated label-free imaging of cells and unstained tissue sections, supporting high-throughput digital pathology. While the current study validates morphological phase contrast on fixed specimens and further demonstrates dry-mass estimation in PBS, future work will extend this framework to time-lapse live-cell imaging to track dynamic dry-mass changes and growth rates, and to further benchmark quantitative accuracy under physiologically relevant conditions.

## Data Availability

The original contributions presented in this study are included in the article. Further inquiries can be directed to the corresponding authors.

## Abbreviations

The following abbreviations are used in this manuscript:

CFA	Color filter array
CMOS	Complementary metal–oxide–semiconductor
DFT	Discrete Fourier transform
DMEM	Dulbecco’s Modified Eagle Medium
FBS	Fetal bovine serum
FFT	Fast Fourier transform
FOV	Field of view
LED	Light-Emitting Diode
GPU	Graphics processing unit
NA	Numerical aperture
PBS	Phosphate-buffered saline
PFA	Paraformaldehyde
PSR	Pixel super-resolution
RGB	Red, green, blue
RMSE	Root mean square error
SBP	Space-bandwidth product
SPD	Spectral power distribution
SSIM	Structural Similarity Index
TIE	Transport of Intensity Equation
USAF	USAF 1951 resolution target
WPGS	Wirtinger Poly-Gradient Solver

## References

[1] Amos B. (2000). Lessons from the history of light microscopy. Nat. Cell Biol..

[2] Stephens D.J., Allan V.J. (2003). Light microscopy techniques for live cell imaging. Science.

[3] Ozcan A., McLeod E. (2016). Lensless imaging and sensing. Annu. Rev. Biomed. Eng..

[4] Greenbaum A., Luo W., Su T.W., Göröcs Z., Xue L., Isikman S.O., Coskun A.F., Mudanyali O., Ozcan A. (2012). Imaging without lenses: Achievements and remaining challenges of wide-field on-chip microscopy. Nat. Methods.

[5] Lukosz W. (1966). Optical systems with resolving powers exceeding the classical limit. J. Opt. Soc. Am..

[6] Bishara W., Sikora U., Mudanyali O., Su T.W., Yaglidere O., Luckhart S., Ozcan A. (2011). Holographic pixel super-resolution in portable lensless on-chip microscopy using a fiber-optic array. Lab Chip.

[7] Isikman S.O., Greenbaum A., Luo W., Coskun A.F., Ozcan A. (2012). Giga-Pixel Lensfree Holographic Microscopy and Tomography Using Color Image Sensors. PLoS ONE.

[8] Mudanyali O., Tseng D., Oh C., Isikman S.O., Sencan I., Bishara W., Oztoprak C., Seo S., Khademhosseini B., Ozcan A. (2010). Compact, light-weight and cost-effective microscope based on lensless incoherent holography for telemedicine applications. Lab Chip.

[9] Ozcan A., Demirci U. (2008). Ultra wide-field lens-free monitoring of cells on-chip. Lab Chip.

[10] Greenbaum A., Luo W., Khademhosseinieh B., Su T.W., Coskun A.F., Ozcan A. (2013). Increased space-bandwidth product in pixel super-resolved lensfree on-chip microscopy. Sci. Rep..

[11] Isikman S.O., Bishara W., Sikora U., Yaglidere O., Yeah J., Ozcan A. (2011). Field-portable lensfree tomographic microscope. Lab Chip.

[12] Ozcan A. (2014). Mobile phones democratize and cultivate next-generation imaging, diagnostics and measurement tools. Lab Chip.

[13] Wu X., Chen Y., Zuo C. (2024). Pixel-super-resolved lensless on-chip microscopy using a color image sensor. Proceedings of the Holography, Diffractive Optics, and Applications XIV.

[14] Lee S.A., Leitao R., Zheng G., Yang S., Rodriguez A., Yang C. (2011). Color capable sub-pixel resolving optofluidic microscope and its application to blood cell imaging for malaria diagnosis. PLoS ONE.

[15] Wu X., Sun J., Chen Y., Wei J., Chen Q., Poon T.C., Gao P., Zuo C. (2024). Wavelength-scanning pixel-super-resolved lens-free on-chip quantitative phase microscopy with a color image sensor. APL Photonics.

[16] Monakhova K., Yanny K., Aggarwal N., Waller L. (2020). Spectral DiffuserCam: Lensless snapshot hyperspectral imaging with a spectral filter array. Optica.

[17] Greenbaum A., Feizi A., Akbari N., Ozcan A. (2013). Wide-field computational color imaging using pixel super-resolved on-chip microscopy. Opt. Express.

[18] Mariën J., Stahl R., Lambrechts A., Van Hoof C., Yurt A. (2020). Color lens-free imaging using multi-wavelength illumination based phase retrieval. Opt. Express.

[19] Wu X., Sun J., Zhang J., Lu L., Chen R., Chen Q., Zuo C. (2021). Wavelength-scanning lensfree on-chip microscopy for wide-field pixel-super-resolved quantitative phase imaging. Opt. Lett..

[20] Guo C., Ma H., Li J., Hong Z., Jiang S., Xiang M., Shao X. (2025). High-throughput pixel-super-resolved coded ptychographic microscopy with a color image sensor. Opt. Express.

[21] Bao P., Zhang F., Pedrini G., Osten W. (2008). Phase retrieval using multiple illumination wavelengths. Opt. Lett..

[22] Zuo C., Li J., Sun J., Fan Y., Zhang J., Lu L., Zhang R., Wang B., Huang L., Chen Q. (2020). Transport of intensity equation: A tutorial. Opt. Lasers Eng..

[23] Bian L., Suo J., Zheng G., Guo K., Chen F., Dai Q. (2015). Fourier ptychographic reconstruction using Wirtinger flow optimization. Opt. Express.

[24] Barer R. (1953). Determination of Dry Mass, Thickness, Solid and Water Concentration in Living Cells. Nature.

[25] Girshovitz P., Shaked N.T. (2012). Generalized cell morphological parameters based on interferometric phase microscopy and their application to cell life cycle characterization. Biomed. Opt. Express.

[26] Horstmeyer R., Ou X., Zheng G., Willems P., Yang C. (2015). Digital pathology with Fourier ptychography. Comput. Med. Imaging Graph.

[27] Bian L., Zheng G., Guo K., Suo J., Yang C., Chen F., Dai Q. (2016). Motion-corrected Fourier ptychography. Biomed. Opt. Express.

